# Emerging Anti-Fouling Methods: Towards Reusability of 3D-Printed Devices for Biomedical Applications

**DOI:** 10.3390/mi9040196

**Published:** 2018-04-20

**Authors:** Eric Lepowsky, Savas Tasoglu

**Affiliations:** 1 Department of Mechanical Engineering, University of Connecticut, Storrs, CT 06269, USA; eric.lepowsky@uconn.edu; 2 Department of Biomedical Engineering, University of Connecticut, Storrs, CT 06269, USA; 3 Institute of Materials Science, University of Connecticut, Storrs, CT 06269, USA; 4 Institute for Collaboration on Health, Intervention, and Policy, University of Connecticut, Storrs, CT 06269, USA; 5 The Connecticut Institute for the Brain and Cognitive Sciences, University of Connecticut, Storrs, CT 06269, USA

**Keywords:** 3D printing, microfluidic chips, anti-fouling, surface coatings

## Abstract

Microfluidic devices are used in a myriad of biomedical applications such as cancer screening, drug testing, and point-of-care diagnostics. Three-dimensional (3D) printing offers a low-cost, rapid prototyping, efficient fabrication method, as compared to the costly—in terms of time, labor, and resources—traditional fabrication method of soft lithography of poly(dimethylsiloxane) (PDMS). Various 3D printing methods are applicable, including fused deposition modeling, stereolithography, and photopolymer inkjet printing. Additionally, several materials are available that have low-viscosity in their raw form and, after printing and curing, exhibit high material strength, optical transparency, and biocompatibility. These features make 3D-printed microfluidic chips ideal for biomedical applications. However, for developing devices capable of long-term use, fouling—by nonspecific protein absorption and bacterial adhesion due to the intrinsic hydrophobicity of most 3D-printed materials—presents a barrier to reusability. For this reason, there is a growing interest in anti-fouling methods and materials. Traditional and emerging approaches to anti-fouling are presented in regard to their applicability to microfluidic chips, with a particular interest in approaches compatible with 3D-printed chips.

## 1. Introduction

Microfluidic devices are widely used in numerous fields. Pertaining specifically to biotechnology, microfluidic devices have been used for cancer screening [1,2,3], microphysiological system engineering [4,5], high-throughput drug testing [6,7], and point-of-care diagnostics [8,9,10]. Within the broad field of microfluidics, novel technologies have been reported, such as paper-based microfluidics and three-dimensional (3D) printed devices. Each of these alternative methods addresses different drawbacks of the conventional soft lithography fabrication of poly(dimethylsiloxane) (PDMS) microfluidic devices. Using paper as a substrate alleviates the need for external pumps, in addition to the added benefits of low-cost fabrication and disposability [11,12,13,14,15,16]. Three-dimensional (3D) printing is a relatively new technique in microfluidics that offers rapid prototyping of a variety of materials, enabling fabrication of high resolution molds, as well as direct fabrication of 3D-printed chips [17,18,19,20,21,22,23,24]. The latter is of particular interest, as microfluidic chips can be designed using computer aided design (CAD) software and printed with high resolution. Compared to the laborious fabrication involved with PDMS chips, 3D-printed chips are low-cost, efficient, and highly customizable. Microfluidic devices, of all forms and methods, are indispensable tools in biomedical engineering. Accordingly, there is a need for reusable, long-term use of microfluidic chips for applications such as long-term health monitoring. Efforts towards designing long-term use microfluidic chips can be aided by the low-cost, high-throughput capabilities offered by 3D-printing.

Fostered by biomedical research, a transformation of the healthcare industry is imminent. There is an unmet need for transformative technologies that are essential for enabling the shift from hospital-centered, reactive care to proactive, person-centered care which focuses on individuals’ well-being [25]. Next-generation technologies are the vital factor in developing affordable and accessible care, while also lowering the costs of healthcare. A promising solution to this challenge is low-cost continuous health monitoring; this approach allows for effective screening, analysis, and diagnosis and facilitates proactive medical intervention. In a study reported by Health Affairs, it was shown that a 90% increase in specific preventative screenings back in 2006 would have saved more than 2 million lives without a significant increase in healthcare costs—in fact, a 0.2% decrease in costs was estimated [26]. Not only does public health benefit, but savings in healthcare costs could be drastically increased by further research focused on the development and implementation of preventative care methods and tools. To support this much-needed approach, there is an impending demand for low-cost, compact, and innovational technologies to perform routine health measurements across the population. Microfluidic devices have a proven record for being effective analytical devices, capable of controlling the flow of fluid samples, containing reaction and detection zones, and displaying results, all within a compact footprint [27,28,29,30]. The next crucial step within microfluidic technologies is to address the need for microfluidic chips capable of long-term use, with a particular interest in designing microfluidic chips that can be embedded within sophisticated medical devices without the need for replacement by the user [31,32].

A primary challenge in producing microfluidic devices for long-term use is the biofouling that often occurs on the surface of integrated channels and features [33,34]. This phenomenon occurs due to surface interactions between the walls of the channels and the biological sample flowing through the channels. As a result, there is an accumulation of unwanted substances on the surface of the microfluidic channels, thereby adversely affecting the performance of the device. Specifically, protein fouling, or nonspecific protein absorption, is the cause of failure for many microfluidic devices [31]. Furthermore, once a protein layer is formed, bacterial attachment and growth of biofilms is facilitated. To address the long-term use of microfluidic devices, there has been a growing research interest in developing new anti-fouling methods and materials [31,32,35,36,37,38,39]. If fouling can be reduced to miniscule levels, a device may be considered reusable for the purpose of point-of-care, long-term health monitoring performed *ex vivo*. Possible solutions for reducing or preventing biofouling have been explored, including dynamic coating and chemical modification. Additionally, with the advent of 3D-printed microfluidic devices, there is a newfound interest in studying the protein absorption of 3D-printed materials [40,41,42]. Directly associated with protein absorption are possible anti-fouling techniques which may be implemented to improve the surface properties of 3D-printed devices. With continued research, these anti-fouling methods applied to 3D-printed microfluidic devices will work towards the future of reusable, long-term use, low-cost, point-of-care biomedical devices. Herein, we assess the reusability of 3D-printed chips for biomedical applications through a review of 3D-printed microfluidic fabrication techniques, 3D-printable materials, and traditional and emerging anti-fouling methods and materials.

## 2. 3D-Printed Microfluidics

### 2.1. Fabrication Methods for 3D-Printed Microfluidics

**Figure 1:**
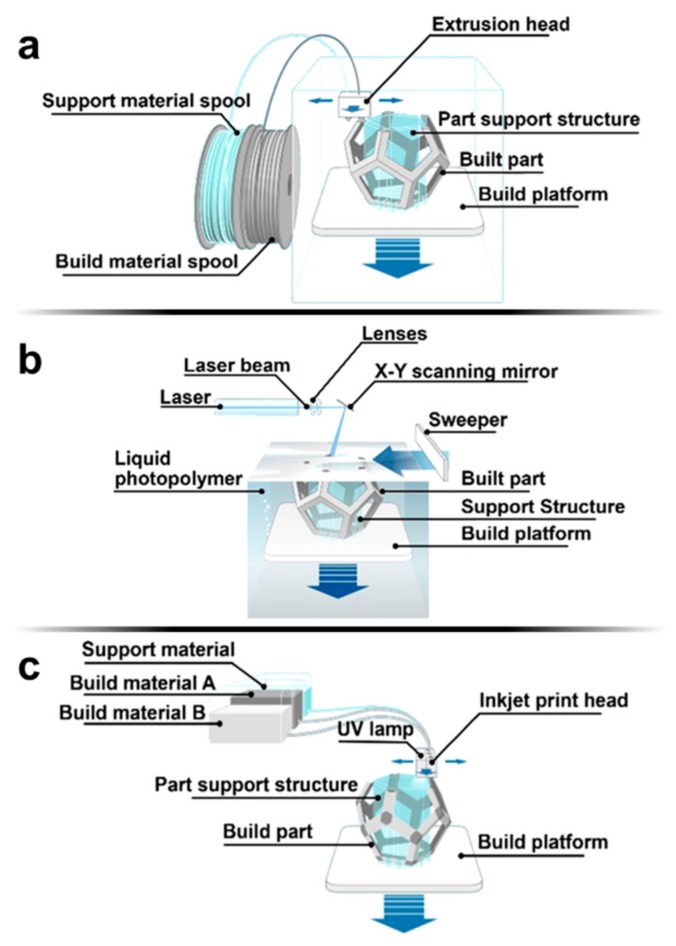
Schematic representation of 3D printing methods commonly applied in the fabrication of microfluidic devices. (**a**) Fused deposition modeling (FDM) [48]: a solid filament is fed from an external spool through the extrusion head, in which the filament is heated and extruded; (**b**) Stereolithography (SLA) [49]: a laser is directed at a scanning mirror which focuses the laser on a pool of photo-sensitive resin; (**c**) Photopolymer inkjet printing [50]: photopolymer material and support material are fed into an inkjet printing head which deposits the material in layers while an attached UV lamp cures the printed material. Illustrations courtesy of [48,49,50].

There are several 3D printing techniques, including the following: fused deposition modeling (FDM), stereolithography (SLA), photopolymer inkjet printing, selection laser sintering (SLS), binder deposition, laminated object manufacturing [19], two-photon polymerization (2PP), and micromirror-controlled projection printing [20]. In FDM, depicted in Figure 1a, a thermoplastic filament is fed through a nozzle [17,19,20,43]. Within the nozzle, rollers force the solid filament through heaters which melt the filament prior to extrusion. The melted filament is then deposited onto the print-bed where it solidifies post-extrusion. SLA 3D printers use light to cure layers of photosensitive resin, as seen in Figure 1b [17,19,20,43]. The resin is cured by photopolymerization caused by a laser or digital projector that is directed towards the surface of the resin by motorized mirrors. SLA offers high resolution, as defined by the size of the laser spot and the type of resin. Photopolymer inkjet printing, modeled in Figure 1c, also prints with a photosensitive polymer, but functions on the concept behind inkjet printing: the liquid photopolymer is deposited onto the print-bed by an inkjet printing head [17,19,20,43,44]. By printing layer-by-layer, each layer is deposited and subsequently cured by UV light. Two powder-based 3D printing methods are SLS and binder jetting. In SLS, a laser is used to thermally bind precursor polymer powders in a layer-by-layer fashion [19,45]. In binder jetting, a liquid adhesive, or binder, is deposited by a print head onto a bed of powder [19,43]. For both SLS and binder deposition, a roller is used to form layers of powder. Another 3D printing method used for microfabrication is 2PP, which uses a near-IR light with high peak power within a UV-photopolymerizable resin. The photo-initiator within the resin absorbs one UV photon and two near-IR photons, causing it to radicalize, thereby breaking its current monomer bonds and forming polymer bonds [19,46,47]. In terms of application in microfluidic device fabrication, FDM, SLA, and photopolymer inkjet printing are the most commonly implemented [18].

### 2.2. Materials for 3D-Printed Microfluidics

Beyond the fabrication methods, another important aspect of 3D-printed microfluidic devices is the material itself. Certain mechanical properties are very relevant to 3D printability, including the elastic modulus and tensile strength of the post-printed material, and the viscosity of the pre-printed material (Table 1) [17]. The elastic modulus is representative of the mechanical relationship between stress and strain. The 3D-printed materials must withstand high internal pressures from the fluid flow; due to the high fluid-induced stress, the resulting degree of strain is an important factor in choosing a material [51]. Likewise, tensile strength also helps to characterize the mechanical properties of the material in terms of the forces it can withstand. Additionally, the viscosity of the material before printing (for resins) is an important factor in determining the printability and resolution of the print. The material must be thin enough (low viscosity) to be precisely deposited in minute quantities but must also be sufficiently thick (high viscosity) such that it does not spread, migrate, or deform during the printing process. Viscosity also plays a role in the ease of cleaning the microfluidic channels post-printing—a lower viscosity is preferred for easier and quicker emptying and rinsing of channels and semi-enclosed geometries.

Additional material properties that are necessary for microfluidics include high optical transparency, hydrophobicity or hydrophilicity, and biocompatibility (Table 1). Optical transparency is extremely useful for microfluidic applications since visual observation and imaging are often necessary. In particular, a transparent microfluidic chip allows for qualitative observations—a visual check on proper fluid flow and reagent interactions—as well as quantitative, through-chip imaging using either visible transmitted light or fluorescence detection. PDMS is widely used for microfluidic device fabrication and has excellent transparency [52]. Several 3D-printable resins have comparable transparency. However, these resins may suffer from fogginess or discoloration over time due to excessive UV-light exposure after fabrication. In some cases, such as for Formlabs clear resin, a thin layer of isopropyl alcohol, oil, or PDMS can be applied to the exterior surfaces of the printed chip to facilitate transparency. Additionally, some commonly used materials in 3D printing exhibit natural fluorescence due to the photo-initiators used for curing, contributing to fluorescent background noise, which could be problematic in fluorescent imaging systems—a challenge which can be mitigated by proper calibration of the imaging process [43,53,54]. Another important property of materials in microfluidics is their intrinsic hydrophobicity or hydrophilicity. Hydrophobic surfaces are more prone to suffering from fouling due to surface interactions, whereas hydrophilic surfaces can facilitate a protective hydration layer [31,55]. Since practically all 3D-printable resins are hydrophobic, either copolymerization, material grafting, or a surface treatment is necessary for anti-fouling purposes. While it should not absorb or react with biological agents in the fluid sample, such as proteins or bacteria, the material should be biocompatible. In biomedical applications, it is likely that living cells may come into contact with the 3D-printed material. Fortunately, the majority of 3D-printable resins are reportedly biocompatible or can be UV-treated to reduce cytotoxicity [20,40,56].

### 2.3. Benefits and Applications of 3D-Printed Microfluidics

Given the variety of fabrication methods and materials available for 3D printing, it is also important to consider the benefits and applications of 3D-printed microfluidics. The conventional method for fabricating microfluidic devices is soft lithography, which involves casting PDMS based on a semi-3D mold [20]. A channel pattern, designed using CAD software, is used to fabricate a master negative-mold. The mold is fabricated, most often by a photoresist process, consisting of spin coating SU-8 on a wafer and exposing it to an ultraviolet source filtered by a photomask. The master mold is then filled with PDMS, which is then allowed to cure. After curing, the PDMS is peeled away from the master, cut into the shape of the desired device, and inlet ports are punched. The cast PDMS is then oxygen plasma treated to improve adhesion to a glass substrate, resulting in the final microfluidic device, in which one of the four channel walls is provided by the glass. This conventional fabrication process is extremely time consuming, involves extensive manual operation, and requires new molds for different designs [75,76,77]. Additionally, the channel height of traditional PDMS microfluidic chips cast using soft lithography, determined by the material properties of the photoresist, is extremely limited. Although photoresist-based molds can have a very high resolution, fabricating complex designs is a tedious and exacting process, requiring photomasks to be perfectly aligned, to expose sequential layers of photoresist [78]. Even with multiple layers of photoresist, three-dimensional PDMS chips are not feasible, as 3D PDMS designs are produced by layering and thus cannot be arbitrarily complex. Three-dimensional (3D) printing offers a solution to these challenges, yet it comes with its own unique set of limitations [19]. The resolution achievable by 3D printing cannot match that of soft lithography. Similarly, the smooth surface of PDMS cannot be recreated by 3D-printed materials. 3D printing can be implemented in two ways: (1) to fabricate a mold, and (2) to directly print the device.

While conventional photoresist-based molds yield smoother surface topology and smaller feature sizes, this method is often economically unfeasible, inaccessible, and involves a lengthy prototyping process [79]. On the other hand, molds can be fabricated by 3D printing, which enables low-cost production of more complex designs. Fabrication of PDMS microfluidic chips using 3D-printed master molds provides many of the advantages of 3D printing fabrication while maintaining the desirable material properties of PDMS, such as biocompatibility and gas permeability [17]. The mold benefits from rapid prototyping techniques, reducing the overall cost and time required for fabrication and enabling a simple method for printing new mold designs. Additionally, 3D-printed molds enable considerably more complex channel geometries: circular cross-section channels [80], non-planar channels such as helices and pillars [81], and even complex intermingling internal patterns supported by sacrificial scaffolds which can be dissolved or melted away post-production [82,83,84].

Three-dimensional (3D) printing can also be implemented to directly fabricate microfluidic devices. Rather than designing a negative-mold for the chip, the chip is printed using the 3D-printed material. This method reduces fabrication costs, increases the speed of production, and is ideal for rapid prototyping and low-cost microfluidic devices. Since the whole device is directly printed, 3D printing eliminates the assembly steps required by conventional molding processes and allows for quick changes to the design, simply requiring a few hours for the new design to print. Another advantage of progressing beyond mold-based fabrication is the ability to produce arbitrarily defined structures in a fully 3D space, with no significant increase in fabrication complexity and time [78,85]. Furthermore, a wide variety of microfluidic components can be fabricated by 3D printing, including passive, which rely on external actuation or capillary forces, and active, which apply energy to manipulate fluid flow components. Examples of passive 3D-printed components include a micromixer, gradient generator, droplet generator, reaction zones, and check valves [17,86,87,88,89]. Active 3D-printed components which have been demonstrated include active valves, flow switches, and pumps [17,54,70].

**Figure 2:**
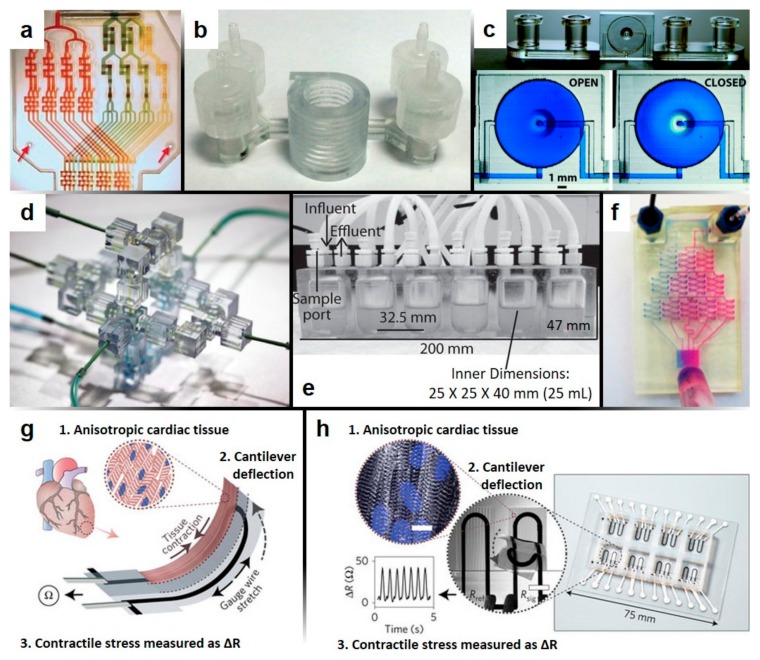
Biomedical applications of 3D-printed microfluidic devices; (**a**) A combinatorial mixer which generates four titrations of two dye solutions and produces combinatorial mixes of the dye titrations to deliver sixteen mixture combinations into separate outlet microchannels (reproduced, with permission, from [98]); (**b**) a helical-shaped 3D microfluidic device with trapezoidal-shaped channels for the detection of pathogenic bacteria by inertial focusing (reproduced, with permission, from [90]); (**c**) automated 3D-printed microfluidic single-valve device. Below are micrographs of the valve unit in its open and closed states (reproduced, with permission, from [70]); (**d**) single-outlet sub-circuit elements are connected to form a four-outlet mixer. Each sub-circuit element is identical, constituted by a single inlet splitter (reproduced, with permission, from [99]); (**e**) example of a simple, high-throughput mini-bioreactor array (MBRA) used for the cultivation of microbial communities (reproduced, with permission, from [19,100]); (**f**) Three-dimensional gradient generator for the mixing of two dyes, consisting of three levels of combining, mixing, and splitting (reprinted, with permission, from [86], Copyright 2014 American Chemical Society); (**g**,**h**) instrumented cardiac microphysiological device fabricated by multi-material 3D printing; (**g**) illustration of the working principles of the microphysiological device; (**h**) images of the fully-printed device (reproduced, with permission, from [101]).

Fully-functional 3D-printed microfluidic chips have also been designed for biomedical applications (Figure 2), such as a chip for inertial focusing and separation of bacteria [90], high-throughput drug transport and cell viability testing [91], electrochemical detection [92], pathogen detection of bacteria and viruses [93,94], biological assays and cell observations [95,96], and cancer assays and studies on cell migration [97]. Figure 2a demonstrates a mixing scheme which has broad applicability to high-throughput combinatorial testing applications, such as drug screening, biochemical assays, lab-on-chip devices, and biosensors [98]. Figure 2b shows an bacteria detection device which separates captured bacteria using inertial focusing [90]. The fabrication of this device’s complex helical geometry and trapezoidal cross-section channels was only made possible by the capabilities of 3D printing. Figure 2c depicts a single-valve 3D-printed microfluidic device which allows for the controlled opening and closing of channels [70]. While soft-lithographic automation generally involves PDMS layering, alignment, and bonding, the pictured device offers a rapid, prototyped alternative, which is completely fabricated by stereolithography. Additionally, two-valve and four-valve switches have also been demonstrated. Such actuated switches can be pivotal in microfluidic sample handling for various assays. Figure 2d shows a three-dimensional microfluidic device built from identical, individual elements [99]. As another example of enhanced control of fluid flow, these modular 3D-printed elements allow for a wide variety of fluidic circuits to be constructed. Figure 2e offers an example of a 3D-printed mini-bioreactor (MBRA) for cultivating fecal microbial communities to study epidemic *Clostridium difficile* strains [100]. The MBRAs used in this study benefitted from the simple, high-throughput fabrication process offered by 3D printing. Figure 2f demonstrates another 3D-printed microfluidic mixing device; however, instead of solely relying on two-dimensional serpentines and splitters, the pictured device utilizes three-dimensional channel patterns to improve the gradient generation [86]. Finally, Figure 2g,h detail a multi-material 3D-printed device for the microphysiological study of cardiac tissues [101]. The functionality of the device relies on contractions of cardiac tissue to deflect a cantilever substrate, thereby stretching an embedded strain gauge which generates a resistance chance proportional to the stress of the tissue (Figure 2g). The device itself was printed with multiple materials to form the cantilever base, strain gauge wire, wire cover, tissue-guiding microfilaments, electrical leads, and surrounding wells. The inset images of Figure 2h show (1) a confocal microscopy image of the immunostained cardiac tissues tested using this device, (2) an image of an active cantilevered device, and (3) an example of the resistance signal from the cantilever deflection. These examples demonstrate the wide applicability of 3D-printed microfluidics to biomedical applications.

## 3. Anti-Fouling Methods and Materials

### 3.1. Traditional Anti-Fouling Methods and Materials

Attributed to the hydrophobicity of PDMS, microfluidic devices fabricated using this traditional material exhibit low wettability and are prone to biofouling from nonspecific protein and analyte absorption and cell and bacterial adhesion [102]. To counteract the fouling, coatings aim to minimize intermolecular forces and interactions between the surface and samples. Pertaining to protein fouling, absorption into the substrate can be due to hydrogen bonding, electrostatic forces, charge-transfers, or hydrophobic interactions [55].

Considerable work has been done to create a hydrophilic PDMS surface with anti-fouling properties [31,36]. Physical methods to accomplish this adaptation rely on changing the state of the PDMS surface via physical processes. For instance, a coating material may be absorbed by the PDMS by hydrophobic or electrostatic interaction. Alternatively, the surface can be treatment-activated by plasma, ozone, or ultraviolet exposure. There are also chemical modification methods that have been studied, such as covalent bonding between a coating material and the PDMS. Both physical and chemical methods have their respective drawbacks—physical modification results in only temporary anti-fouling, with the effectiveness decreasing over time, while chemical modification requires complex processes with several reagents and procedural steps. Consequently, these methods may not be ideal for commercialization, particularly for fabricating long-term use or low-cost devices, since they are often ephemeral and laborious. Polymer chain length is an important factor when considering anti-fouling mechanisms [31]. Protein resistance can be achieved by a hydration layer of short chain length polymers, which forms due to the hydrophilicity of the short chains. In addition to forming a hydrophilic hydration layer, long chain length materials have the added benefit of steric repulsion from the flexible polymer chains. When tuning the protein resistance of a material, longer chain lengths are therefore preferred since they exhibit both a hydration layer and steric repulsion. Beyond hydrophilicity, anti-fouling mechanisms should be electrically neutral and should only have hydrogen bond acceptors—no hydrogen bond donors [103].

A widely used anti-fouling surface treatment is a poly(ethylene oxide) (PEO) or poly(ethylene glycol) (PEG) coating. PEO and PEG are hydrophilic and nontoxic [104,105]. They can be added as a coating on PDMS by physical or chemical absorption, direct covalent attachment, or graft copolymerization. The addition of PEG chains to the surface of the microfluidic channels offers anti-fouling capabilities due to a hydration layer and steric repulsion, the strength of which is dependent on factors such as surface density and the length of the PEG chains [106,107,108,109,110]. Another class of materials that has been demonstrated for anti-fouling purpose is zwitterions. Polyzwitterion-based coatings have an equal amount of positively and negatively charged groups and thus exhibit electrical neutrality, which is a desirable characteristic [31]. Zwitterionic materials are able to form thick hydration layers by bonding with water molecules, forming a shield across the surface to prevent protein absorption [111,112,113,114,115,116]. Other types of anti-fouling coatings to prevent the absorption of nonspecific proteins include saccharide-based coatings [117,118,119,120], polyhydroxy-polymer-based coatings [121,122], amide-containing-hydrophilic-polymer-based coatings [36,123,124], and fluorinated-polymer-based coatings [125,126,127,128].

### 3.2. Emerging Anti-Fouling Methods and Materials

The numerous methods proposed for anti-fouling of PDMS suggest that reusable microfluidic devices are plausible. However, other materials and methods are needed for anti-fouling coatings of 3D-printed microfluidic devices. Novel advancements from the Wyss Institute at Harvard University have yielded SLIPS (Slippery Liquid-Infused Porous Surfaces). SLIPS is an ultra-repellent, self-healing, transparent surface coating for industrial and medical materials [129]. While most repellent surface materials can repel aqueous substances, they often fail to repel other liquids, fail or degrade under physical stress, cannot self-heal after repeated use, and may be very costly to implement [129,130,131]. The SLIPS technology relies on nano- and micro-structured porous material infused with lubricating fluid [129]. The commonality between the traditional methods and materials described above and SLIPS is the implementation of a lubrication or hydration layer. High optical transparency and compatibility with a myriad of substrates are what differentiate SLIPS from other coatings. These features also make SLIPS an ideal anti-fouling material for 3D-printed microfluidic chips for biomedical applications, in addition to a variety of previously reported applications [130,131,132,133].

**Figure 3:**
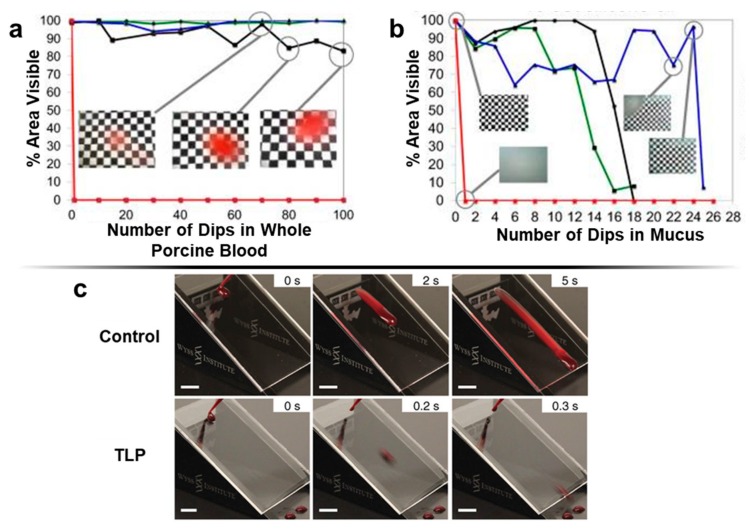
Emerging anti-fouling methods and materials: SLIPS (Slippery Liquid-Infused Porous Surfaces). (**a**,**b**) Evolution of the reduction in the visible area of an endoscope surface coated by SLIPS as a function of the number of dips. Silicone oil of low viscosity (10 cSt) was used as the lubricating liquid. Red corresponds to an uncoated endoscope, which fails immediately after a single dip. Green, blue, and black correspond to three replicates of SLIPS coated endoscopes; (**a**) endoscope dipped in whole porcine blood. Inset images show the visibility of the field of view at 70, 08, and 100 dips for the poorest performing sample; (**b**) endoscope dipped in mucus. Insets show the visibility of a coated endoscope after repeated dips compared to an untreated endoscope (reproduced, with permission, from [37]). (**c**) Comparison of the repellency of a tethered-liquid perfluorocarbon (TLP) treated surface to an untreated surface (reproduced, with permission, from [35]).

As a transparent, anti-fouling material, SLIPS was demonstrated for implementation on an endoscope lens to prevent vision loss after repeated submersions in blood and mucus [37]. The coating exhibited conformability, mechanical adhesion, high transparency, biocompatibility, and high resistance to fogging and fouling by bodily fluids (Figure 3a,b) [134,135]. These material characteristics were attributed to the liquid-infused structure of the coating, which consisted of a porous silica particle network infiltrated with various oils, such as silicone oil which is already used in a variety of medical applications due to its biocompatibility and proven clinical performance [37,130,136,137,138]. By applying the coating to the lens, cleaning procedures in between uses of the endoscope were unnecessary or, in the worst cases, reduced to ten-to-fifteen times shorter than for an untreated lens. Additionally, the surface coating maintained a clear view through the lens, which is also extremely important for 3D-printed microfluidic chips for diagnostic applications, as diagnostics often rely on through-chip imaging.

As opposed to the stationary lens of an endoscope, in an environment with interfaces under flow, such as the channels of a microfluidic chip, it is also important to consider the stability and longevity of immobilized hydration layers as surface coatings. The surface hydration layer provides a low-adhesion interface that deters the attachment of fouling materials. However, as fouling material-laden fluid flows over the hydration layer, there is an increase in the instability of the interface [139]. Experimental results and mathematical modeling by Howell et al. provided several important findings: (1) initial conditioning to flush out excess lubricant from the surface reduces further losses; (2) surface structure and lubricant viscosity do not cause significant differences in performance; and (3) the formation of an air–water interface within the channel causes disruptions in the lubricant layer.

SLIPS technology has also been applied as a coating for medical materials that can effectively suppress thrombosis, even under high-pressure and high-shear flow [35]. An anti-thrombogenic surface coating was formed by covalently binding a flexible tethered perfluorocarbon (TP) layer on the material surface, which was then coated by a layer of a liquid perfluorocarbon (LP). The resulting bilayer is referred to as a tethered-liquid perfluorocarbon (TLP) surface. The TLP is capable of repelling blood and preventing the adhesion of blood components and bacteria, thereby reducing thrombosis without the need for anti-coagulants (Figure 3c). Even when subjected to a flowing fluid, the TP maintains the LP hydration layer. The TLP is also simple to fabricate—virtually any substrate can be modified by low-plasma surface modification, a procedure which is commonly used for the commercial modification of materials and is independent of surface geometry and properties [140]. The TLP coating is ideal for treating the surfaces of 3D-printed microfluidic channels as it is compatible with a large variety of materials and exhibits anti-thrombogenic, anti-bacterial, and anti-fouling properties.

Another lubricant-infused coating that has been applied to polymeric biomedical devices is a new, advanced type of the traditional fluorinated-polymer-based coatings. Badv et al. recently demonstrated an omniphobic, lubricant-infused coating which was applied to catheters to prevent thrombosis [141]. The proposed omniphobic coating functioned on a similar liquid lubricant concept as SLIPS—a biocompatible liquid lubricant is held to the surface of the catheter to form an anti-fouling coating. However, instead of forming a hydrophilic hydration layer as SLIPS aims to accomplish, the coating developed by Badv et al. was based on a self-assembled monolayer (SAM) of hydrophobic organosilanes which attenuates clotting. Recently, omniphobic, lubricant-infused coatings have been developed as a new class of coatings created by tethering biocompatible, perfluorocarbon lubricants onto SAMs of hydrophobic organosilanes [35,130]. In greater detail, fluorous molecules can be physically adsorbed onto fluorous-containing surfaces, facilitated by the strong intermolecular interaction between the fluorinated lubricant and the fluorosilane layer. Such omniphobic coatings are excellent for resisting blood clot formation and are more effective than traditional anti-fouling methods, such as PEG grafting, for blocking non-specific adhesion of cells and bacteria [142,143]. In addition to the great effectiveness of omniphobic coatings, they are stable and durable when exposed to shear stress [35,139].

A feature that sets the work of Badv et al. apart from other traditional omniphobic, fluorinated-polymer-based coatings is the method of application. The most common technique for applying SAMs of fluorine-based silanes is liquid phase deposition (LPD). LPD has a few notable drawbacks and limitations. LPD is not commercially feasible due to the high volume of solvent waste that it produces which is harmful to the environment [144]. Another limitation is that the self-polymerization of silanes in the liquid may prevent homogenous silane layer formation [145]. Additionally, LPD-treated surfaces are likely to be exposed to impurities and reaction side products which may have detrimental effects on the surface itself [144]. Conversely, the proposed surface coating was applied via chemical vapor deposition (CVD), which has the following benefits: a simplified application process, a lesser effect on the topography of the receiving substrate, and greater effectiveness. CVD was performed by oxygen plasma treatment of the catheters, followed by silanization, and completed by the addition of a biocompatible liquid lubricant.

### 3.3. Anti-Fouling Methods Applied to 3D-Printed Materials

Biofouling from nonspecific protein and analyte absorption and cell and bacterial adhesion is not a detriment of only PDMS; biofouling can occur on the surfaces of all microfluidic chips, of all materials. While extensive work has been conducted to address the anti-fouling of PDMS, disproportionately less work has been reported for 3D printing. Nonetheless, recent studies and reviews have considered the biofouling of 3D-printed materials, demonstrating its significance and importance in the field. The objective of a study done by Siddiqui et al. was to propose a strategy for developing, characterizing, and testing 3D-printed feed spacers, both numerically by membrane fouling simulator (MFS) studies and experimentally [42]. Although the biofouling of the 3D-printed device was not directly assessed, the results showed that a 3D-printed feed spacer was similar. Feed spacers are commonly used devices in spiral-wound reverse osmosis and nanofiltration membrane systems to enhance water mixing and to reduce biofouling. As reported by Baker et al., the surface of feed spacers is prone to the initial deposition of fouling which can accumulate and spread [146]. Van Paassen et al. then showed that biofouling accumulated on feed spacers causes an exponential increase in the pressure drop across the membrane module containing the feed spacer [147]. To remedy the biofouling of feed spacers, various solutions have been proposed [148,149]. More recently, researchers have focused on the surface chemistry of the feed spacers. Multiple studies have considered anti-fouling coatings; however, these are more likely to wear out over time [150,151,152,153,154]. The solution proposed by Siddiqui et al. considered the native anti-fouling properties of 3D-printed materials. Their findings demonstrated the benefits of 3D-printing—feed spacers can be designed and printed with complex geometries to reduce the effects of biofouling. This studied demonstrates an example of the importance and application of studying the fouling of 3D-printed materials.

In reporting on the 3D printing of biopolymers for tissue engineering applications, Li et al. discussed the biocompatibility of common 3D-printed materials, such as polycaprolactone (PCL), poly-lactic acid (PLA), and acrylonitrile butadiene styrene (ABS) [41]. While PCL and PLA are natively biocompatible, ABS is not as ideally suited for biomedical applications. For this reason, there is considerable interest in possible methods for modifying the surface of printed ABS to render it hydrophilic and biocompatible. A traditional anti-fouling method which is compatible with a wide variety of materials, including ABS and other 3D-printed materials, is surface modification by the grafting of PEG [41,110]. As an alternative, McCullough and Yadavlli utilized an acetone solution to transform a porous ABS device fabricated by fused deposition modeling into a water-tight microfluidic device [155]. The acetone-treated microfluidic device retained the structural fidelity of microstructures as small as 250 µm, while benefiting from improved water impermeability, hydrophilicity, and biocompatibility. In addition to the acetone soaking, a traditional process of photo-induced PEG grafting was performed to further facilitate the formation of a stable, biocompatible surface by reducing biofouling behavior [155].

Anti-fouling methods have also been applied to 3D-printed molds for microfluidic device fabrication. Villegas et al. utilized omniphobic, lubricant-infused coatings, similar to the previously coating implemented by Badv et al., to fabricate 3D-printed molds for fabricating smooth PDMS microfluidic channels [79,141]. While the use of 3D-printed molds for casting PDMS microfluidic devices has been previously reported and described above, methods to improve the surface topology of the mold have not been previously studied [81,82]. The surface topology of a 3D-printed mold is likely to exhibit high variability and roughness, thereby increasing the difficulty of creating smooth, defined microfluidic channels from the mold [80]. In the recent study by Villegas et al., they addressed this challenge by investigating the use of lubricant-infused surfaces as a method for decreasing surface roughness—in addition to the more common use of lubricant-infusion for creating omniphobic slippery surfaces, which can be used in a multitude of applications including anti-biofouling. In this particular study, the mold was 3D-printed using a multi-jet modeling printer equipped with high-resolution nozzles [156]. The mold was then sonicated in ethanol and oxygen plasma treated before a self-assembled monolayer of fluorosilane was applied by chemical vapor deposition. Finally, a fluorocarbon lubricant was applied to form the smooth, lubricant-infused interface. This study, therefore, demonstrated the compatibility of 3D-printed material with omniphobic lubricant-infused coatings applied via chemical vapor deposition.

## 4. Conclusions

Microfluidic devices are invaluable tools in biomedical engineering. Microfluidics have a proven record of being effective analytical and diagnostic devices. Presently, there is an ongoing evolution in the field of microfluidics as researchers explore 3D printing as a viable fabrication method. 3D printing is revolutionizing the fabrication workflow of microfluidics. As opposed to the time- and resource-intensive process of soft lithography for PDMS chip fabrication, 3D printing offers a low-cost, customizable platform for the direct fabrication of microfluidic chips. There are several 3D printing methods, of which fused deposition modeling, stereolithography, and photopolymer inkjet printing are applicable to 3D-printed microfluidic chips. Additionally, many materials are offered that may be compatible, boasting features such as high mechanical strength post-printing, low viscosity pre-printing, optical transparency, and biocompatibility.

The diagnostic capabilities of microfluidic chips and the benefits of 3D printing can be leveraged to address the unmet need for the long-term use of microfluidic chips for biomedical applications, such as for long-term health monitoring. Long-term, routine health monitoring has the capacity to improve public health while also reducing healthcare costs. A noteworthy obstacle in developing long-term diagnostic microfluidic chips is the fouling that is common on the surfaces of integrated microchannels and features. Similar to traditional PDMS, 3D-printed resins are most often hydrophobic, which means they are prone to surface interactions between the walls of the channels and the biological sample flowing through the channels. The resulting accumulation of unwanted substances on the surface of the microfluidic channels adversely affects the performance of the device. To combat fouling, there has been a growing research interest in developing new anti-fouling methods and materials. Traditional anti-fouling relies on chemical and/or physical surface modification. As an emerging solution, SLIPS (Slippery Liquid-Infused Porous Surfaces) offers a transparent, durable and robust surface treatment that can be applied to practically any surface, including the channels of a 3D-printed microfluidic chip. Continued research and characterization will result in reusable 3D-printed microfluidic chips that are low-cost, robust, transparent, and biocompatible.

## Figures and Tables

**Table 1:** Materials for 3D printing.

	3D Printing Properties			Important Properties for Microfluidics		
Material/Example	Elastic Modulus (GPa)	Tensile Strength (MPa)	Viscosity (cps)	Optical Transparency	Hydro-Phobicity/Philicity	Bio-Compatibility
Poly(dimethylsiloxane) (PDMS) (for comparison) [52,57]	0.00132–0.00297	3.51–7.65	N/A	High transparency (standard)	Hydrophobic	Biocompatible
MakerBot polylactic acid (PLA) [40,58,59]	3.368	56.6	N/A	Semi-transparent	Hydrophobic, easily modified	Biocompatible, biodegradable
MakerBot acrylonitrile butadiene styrene (ABS) [40,58]	1.807	28.5	N/A	Opaque	Hydrophobic	Biocompatible
Formlabs proprietary methacrylate [40,43,60,61,62]	2.7	61.5	850–900	Transparent, discolors	Hydrophobic	N/A
Asiga PlasCLEAR polypropylene/ABS [43,63,64]	N/A	52.6	342	Semi-transparent	N/A	N/A
Stratasys Object acrylates and acrylics [19,43,65]	2–3	50–65	N/R	Transparent, discolors	Hydrophobic	Biocompatible
3DSystems VisiJet Clear Class [40,66,67,68,69]	0.866–2.168	20.5–49	150–260	Semi-transparent	N/A	Biocompatible
Somos WaterShed XC [19,40,43,70,71,72]	2.77	50.4	260	Transparent, discolors	Hydrophobic	Biocompatible
MiiCraft acrylates (BV-007 Clear Resin) [43,73]	N/A	N/A	N/A	Semi-transparent	N/A	Biocompatible available
DWS Lab Vitra 429 & DS3000 [74]	1.38	32–35	600–850	Transparent	N/A	Short-term biocompatible

## References

[1] Wankhede S.P., Du Z., Berg J.M., Vaughn M.W., Dallas T., Cheng K.H., Gollahon L. (2006). Cell detachment model for an antibody-based microfluidic cancer screening system. Biotechnol. Prog..

[2] Leoncini E., Ricciardi W., Cadoni G., Arzani D., Petrelli L., Paludetti G., Brennan P., Luce D., Stucker I., Matsuo K. (2014). Lab-on-a-chip for oral cancer screening and diagnosis. Head Neck.

[3] Kim L. (2013). Overview of the Microfluidic Diagnostics Commercial Landscape.

[4] Srinivasan V., Pamula V.K., Fair R.B. (2004). An integrated digital microfluidic lab-on-a-chip for clinical diagnostics on human physiological fluids. Lab Chip.

[5] Hsu Y.-H., Moya M.L., Hughes C.C.W., George S.C., Lee A.P. (2013). A microfluidic platform for generating large-scale nearly identical human microphysiological vascularized tissue arrays. Lab Chip.

[6] Toh Y.-C., Lim T.C., Tai D., Xiao G., van Noort D., Yu H. (2009). A microfluidic 3D hepatocyte chip for drug toxicity testing. Lab Chip.

[7] Yu L., Chen M.C.W., Cheung K.C. (2010). Droplet-based microfluidic system for multicellular tumor spheroid formation and anticancer drug testing. Lab Chip.

[8] Chin C.D., Linder V., Sia S.K. (2012). Commercialization of microfluidic point-of-care diagnostic devices. Lab Chip.

[9] Sia S.K., Kricka L.J. (2008). Microfluidics and point-of-care testing. Lab Chip.

[10] Foudeh A.M., Fatanat Didar T., Veres T., Tabrizian M. (2012). Microfluidic designs and techniques using lab-on-a-chip devices for pathogen detection for point-of-care diagnostics. Lab Chip.

[11] Amin R., Ghaderinezhad F., Li L., Lepowsky E., Yenilmez B., Knowlton S., Tasoglu S. (2017). Continuous-Ink, Multiplexed Pen-Plotter Approach for Low-Cost, High-Throughput Fabrication of Paper-Based Microfluidics. Anal. Chem..

[12] Cate D.M., Adkins J.A., Mettakoonpitak J., Henry C.S. (2015). Recent developments in paper-based microfluidic devices. Anal. Chem..

[13] Martinez A.W., Phillips S.T., Whitesides G.M., Carrilho E. (2010). Diagnostics for the developing world: Microfluidic paper-based analytical devices. Anal. Chem..

[14] Yetisen A.K., Akram M.S., Lowe C.R. (2013). Paper-based microfluidic point-of-care diagnostic devices. Lab Chip.

[15] Lepowsky E., Ghaderinezhad F., Knowlton S., Tasoglu S. (2017). Paper-based assays for urine analysis. Biomicrofluidics.

[16] Ghaderinezhad F., Amin R., Temirel M., Yenilmez B., Wentworth A., Tasoglu S. (2017). High-throughput rapid-prototyping of low-cost paper-based microfluidics. Sci. Rep..

[17] Amin R., Knowlton S., Hart A., Yenilmez B., Ghaderinezhad F., Katebifar S., Messina M., Khademhosseini A., Tasoglu S. (2016). 3D-printed microfluidic devices. Biofabrication.

[18] Amin R., Joshi A., Tasoglu S. (2017). Commercialization of 3D-printed microfluidic devices. J. 3D Print. Med..

[19] Au A.K., Huynh W., Horowitz L.F., Folch A. (2016). 3D-Printed Microfluidics. Angew. Chemie Int. Ed..

[20] Ho C.M.B., Ng S.H., Li K.H.H., Yoon Y.-J. (2015). 3D printed microfluidics for biological applications. Lab Chip.

[21] Sochol R.D., Sweet E., Glick C.C., Wu S., Yang C., Restaino M., Lin L. (2018). Microelectronic engineering 3D printed microfluidics and microelectronics. Microelectron. Eng..

[22] Knowlton S., Yu C.H., Ersoy F., Emadi S., Khademhosseini A., Tasoglu S. (2016). 3D-printed microfluidic chips with patterned, cell-laden hydrogel constructs. Biofabrication.

[23] Knowlton S., Yenilmez B., Tasoglu S. (2016). Towards Single-Step Biofabrication of Organs on a Chip via 3D Printing. Trends Biotechnol..

[24] Knowlton S., Joshi A., Syrrist P., Coskun A.F., Tasoglu S. (2017). 3D-Printed Smartphone-Based Point of Care Tool for Fluorescence- and Magnetophoresis-Based Cytometry. Lab Chip.

[25] National Science Foundation Smart and Connected Health (SCH). https://www.nsf.gov/pubs/2016/nsf16601/nsf16601.htm.

[26] Ward B.W., Clarke T.C., Nugent C.N., Schiller J.S. Early Release of Selected Estimates Based on Data From the 2015 National Health Interview Survey (05/2016). https://www.cdc.gov/nchs/data/nhis/earlyrelease/earlyrelease201605.pdf.

[27] Ashraf M.W., Tayyaba S., Afzulpurkar N. (2011). Micro Electromechanical Systems (MEMS) based microfluidic devices for biomedical applications. Int. J. Mol. Sci..

[28] Jivani R.R., Lakhtaria G.J., Patadiya D.D., Patel L.D., Jivani N.P., Jhala B.P. (2016). Biomedical microelectromechanical systems (BioMEMS): Revolution in drug delivery and analytical techniques. Saudi Pharm. J..

[29] Fujii T. (2002). PDMS-based microfluidic devices for biomedical applications. Microelectron. Eng..

[30] Dario P., Carrozza M.C., Benvenuto A., Menciassi A. (2000). Micro-systems in biomedical applications. J. Micromech. Microeng..

[31] Zhang H., Chiao M. (2015). Anti-fouling coatings of poly(dimethylsiloxane) devices for biological and biomedical applications. J. Med. Biol. Eng..

[32] Picher M.M., Küpcü S., Huang C.-J., Dostalek J., Pum D., Sleytr U.B., Ertl P. (2013). Nanobiotechnology advanced antifouling surfaces for the continuous electrochemical monitoring of glucose in whole blood using a lab-on-a-chip. Lab Chip.

[33] Wong I., Ho C.M. (2009). Surface molecular property modifications for poly(dimethylsiloxane) (PDMS) based microfluidic devices. Microfluid. Nanofluid..

[34] Goyanes A., Buanz A.B.M., Hatton G.B., Gaisford S., Basit A.W. (2015). 3D printing of modified-release aminosalicylate (4-ASA and 5-ASA) tablets. Eur. J. Pharm. Biopharm..

[35] Leslie D.C., Waterhouse A., Berthet J.B., Valentin T.M., Watters A.L., Jain A., Kim P., Hatton B.D., Nedder A., Donovan K. (2014). A bioinspired omniphobic surface coating on medical devices prevents thrombosis and biofouling. Nat. Biotechnol..

[36] Tu Q., Wang J.C., Liu R., He J., Zhang Y., Shen S., Xu J., Liu J., Yuan M.S., Wang J. (2013). Antifouling properties of poly(dimethylsiloxane) surfaces modified with quaternized poly(dimethylaminoethyl methacrylate). Colloids Surf. B Biointerfaces.

[37] Sunny S., Cheng G., Daniel D., Lo P., Ochoa S., Howell C., Vogel N., Majid A., Aizenberg J. (2016). Transparent antifouling material for improved operative field visibility in endoscopy. Proc. Natl. Acad. Sci. USA.

[38] Defense Sciences Office DARPA DARPA-RA-17-01: Young Faculty Award. https://www.darpa.mil/attachments/DARPA-RA-17-01FAQ11-17-17.pdf.

[39] Wan A.M.D., Devadas D., Young E.W.K. (2017). Recycled polymethylmethacrylate (PMMA) microfluidic devices. Sens. Actuators B Chem..

[40] Bhattacharjee N., Urrios A., Kang S., Folch A. (2016). The upcoming 3D-printing revolution in microfluidics. Lab Chip.

[41] Li X., Cui R., Sun L., Aifantis K.E., Fan Y., Feng Q., Cui F., Watari F. (2014). 3D-printed biopolymers for tissue engineering application. Int. J. Polym. Sci..

[42] Siddiqui A., Farhat N., Bucs S.S., Linares R.V., Picioreanu C., Kruithof J.C., Van Loosdrecht M.C.M., Kidwell J., Vrouwenvelder J.S. (2016). Development and characterization of 3D-printed feed spacers for spiral wound membrane systems. Water Res..

[43] Waheed S., Cabot J.M., Macdonald N.P., Lewis T., Guijt R.M., Paull B., Breadmore M.C. (2016). 3D printed microfluidic devices: Enablers and barriers. Lab Chip.

[44] Singh M., Haverinen H.M., Dhagat P., Jabbour G.E. (2010). Inkjet printing-process and its applications. Adv. Mater..

[45] Mazzoli A. (2013). Selective laser sintering in biomedical engineering. Med. Biol. Eng. Comput..

[46] Xing J.-F., Zheng M.-L., Duan X.-M. (2015). Two-photon polymerization microfabrication of hydrogels: an advanced 3D printing technology for tissue engineering and drug delivery. Chem. Soc. Rev..

[47] Maruo S., Nakamura O., Kawata S. (1997). Three-dimensional microfabrication with two-photon-absorbed photopolymerization. Opt. Lett..

[48] Additively Fused Deposition Modeling (FDM). https://www.additively.com/en/learn-about/fused-deposition-modeling.

[49] Additively Stereolithography (SL). https://www.additively.com/en/learn-about/stereolithography.

[50] Additively Photopolymer Jetting (PJ). https://www.additively.com/en/learn-about/photopolymer-jetting.

[51] Stone H.A., Stroock A.D., Ajdari A. (2004). Engineering Flows in Small Devices: Microfluidics Toward a Lab-on-a-Chip. Annu. Rev. Fluid Mech..

[52] Mata A., Fleischman A.J., Roy S. (2005). Characterization of polydimethylsiloxane (PDMS) properties for biomedical micro/nanosystems. Biomed. Microdevices.

[53] Qaderi K. (2015). Polyethylene Glycol Diacrylate (PEGDA) Resin Development for 3D-Printed Microfluidic Devices. Master’s Thesis.

[54] Rogers C.I., Qaderi K., Woolley A.T., Nordin G.P. (2015). 3D printed microfluidic devices with integrated valves. Biomicrofluidics.

[55] Liu J., Lee M.L. (2006). Permanent surface modification of polymeric capillary electrophoresis microchips for protein and peptide analysis. Electrophoresis.

[56] Oskui S.M., Diamante G., Liao C., Shi W., Gan J., Schlenk D., Grover W.H. (2016). Assessing and Reducing the Toxicity of 3D-Printed Parts. Environ. Sci. Technol. Lett..

[57] Johnston I.D., McCluskey D.K., Tan C.K.L., Tracey M.C. (2014). Mechanical characterization of bulk Sylgard 184 for microfluidics and microengineering. J. Micromech. Microeng..

[58] Tymrak B.M., Kreiger M., Pearce J.M. (2014). Mechanical properties of components fabricated with open-source 3-D printers under realistic environmental conditions. Mater. Des..

[59] Ramot Y., Haim-Zada M., Domb A.J., Nyska A. (2016). Biocompatibility and safety of PLA and its copolymers. Adv. Drug Deliv. Rev..

[60] Ma Y., Cao X., Feng X., Ma Y., Zou H. (2007). Fabrication of super-hydrophobic film from PMMA with intrinsic water contact angle below 90°. Polymer (Guildf.).

[61] Brandhoff L., van den Driesche S., Lucklum F., Vellekoop M.J. (2015). Creation of hydrophilic microfluidic devices for biomedical application through stereolithography. SPIE Microtechnol..

[62] Formlabs Formlabs Clear Safety Data Sheet. https://formlabs.com/media/upload/Clear-SDS_u324bsC.pdf.

[63] Takenaga S., Schneider B., Erbay E., Biselli M., Schnitzler T., Schöning M.J., Wagner T. (2015). Fabrication of biocompatible lab-on-chip devices for biomedical applications by means of a 3D-printing process. Phys. Status Solidi Appl. Mater. Sci..

[64] Asiga PlasCLEAR Technical Datasheet. https://www.asiga.com/media/main/files/materials/PlasCLEAR_us_en.pdf.

[65] Stratasys Stratasys PolyJet Materials Material Safety Data Sheets. http://www.stratasys.com/materials/material-safety-data-sheets/polyjet/transparent-materials.

[66] Martino C., Berger S., Wootton R.C.R., deMello A.J. (2014). A 3D-printed microcapillary assembly for facile double emulsion generation. Lab Chip.

[67] 3D System Material Selection Guide for Stereolithography—SLA. https://www.3dsystems.com/sites/default/files/2017-06/3D-Systems_SLS_MaterialSelectionGuide_USEN_2017.06.28_WEB.pdf.

[68] 3D Systems VisiJet S300 Specification Sheet. http://infocenter.3dsystems.com/projetmjp3600/user-guide/introduction/important-safety-information/visijet®-s300-specification-sheet.

[69] 3D Systems VisiJet MP200, VisiJet M3 StonePlast Safety Data Sheet. http://infocenter.3dsystems.com/materials/sites/default/files/sds-files/professional/24156-s12-02-asds_ghsenglishvisijet_mp200_and_m3_stoneplast.pdf.

[70] Au A.K., Bhattacharjee N., Horowitz L.F., Chang T.C., Folch A. (2015). 3D-Printed Microfluidic Automation. Lab Chip.

[71] Wood S., Krishnamurthy N., Santini T., Raval S., Farhat N., Holmes J.A., Ibrahim T.S. (2017). Design and fabrication of a realistic anthropomorphic heterogeneous head phantom for MR purposes. PLoS ONE.

[72] Proto Labs Product Data: Somos WaterShed XC 11122. https://www.protolabs.com/media/1010884/somos-watershed-xc-11122.pdf.

[73] MiiCraft MiiCraft-Technical Data Sheet: BV-007 Clear Resin. http://www.miicraft.com/support/.

[74] DWS Lab Materials Range. http://www.dwslab.com/materials-range/?v=7516fd43adaa.

[75] Choudhury D., Mo X., Iliescu C., Tan L.L., Tong W.H., Yu H. (2011). Exploitation of physical and chemical constraints for three-dimensional microtissue construction in microfluidics. Biomicrofluidics.

[76] Waldbaur A., Rapp H., Länge K., Rapp B.E. (2011). Let there be chip—Towards rapid prototyping of microfluidic devices: One-step manufacturing processes. Anal. Methods.

[77] O’Neill P.F., Ben Azouz A., Vázquez M., Liu J., Marczak S., Slouka Z., Chang H.C., Diamond D., Brabazon D. (2014). Advances in three-dimensional rapid prototyping of microfluidic devices for biological applications. Biomicrofluidics.

[78] Spivey E.C., Xhemalce B., Shear J.B., Finkelstein I.J. (2014). 3D-printed microfluidic microdissector for high-throughput studies of cellular aging. Anal. Chem..

[79] Villegas M., Cetinic Z., Shakeri A., Didar T.F. (2018). Fabricating smooth PDMS microfluidic channels from low-resolution 3D printed molds using an omniphobic lubricant-infused coating. Anal. Chim. Acta.

[80] Hwang Y., Seo D., Roy M., Han E., Candler R.N., Seo S. (2016). Capillary Flow in PDMS Cylindrical Microfluidic Channel Using 3-D Printed Mold. J. Microelectromech. Syst..

[81] Hwang Y., Paydar O.H., Candler R.N. (2015). 3D printed molds for non-planar PDMS microfluidic channels. Sensors Actuators, A Phys..

[82] Saggiomo V., Velders A.H. (2015). Simple 3D Printed Scaffold-Removal Method for the Fabrication of Intricate Microfluidic Devices. Adv. Sci..

[83] Gelber M.K., Bhargava R. (2015). Monolithic multilayer microfluidics via sacrificial molding of 3D-printed isomalt. Lab Chip.

[84] He Y., Qiu J., Fu J., Zhang J., Ren Y., Liu A. (2015). Printing 3D microfluidic chips with a 3D sugar printer. Microfluid. Nanofluid..

[85] Bonyár A., Sántha H., Ring B., Varga M., Kovács J.G., Harsányi G. (2010). 3D Rapid Prototyping Technology (RPT) as a powerful tool in microfluidic development. Procedia Eng..

[86] Shallan A.I., Smejkal P., Corban M., Guijt R.M., Breadmore M.C. (2014). Cost-effective three-dimensional printing of visibly transparent microchips within minutes. Anal. Chem..

[87] Kitson P.J., Rosnes M.H., Sans V., Dragone V., Cronin L. (2012). Configurable 3D-Printed millifluidic and microfluidic “lab on a chip” reactionware devices. Lab Chip.

[88] Donvito L., Galluccio L., Lombardo A., Morabito G., Nicolosi A., Reno M. (2015). Experimental validation of a simple, low-cost, T-junction droplet generator fabricated through 3D printing. J. Micromech. Microeng..

[89] Comina G., Suska A., Filippini D. (2015). 3D printed unibody lab-on-a-chip: Features survey and check-valves integration. Micromachines.

[90] Lee W., Kwon D., Choi W., Jung G.Y., Au A.K., Folch A., Jeon S. (2015). 3D-Printed microfluidic device for the detection of pathogenic bacteria using size-based separation in helical channel with trapezoid cross-section. Sci. Rep..

[91] Anderson K.B., Lockwood S.Y., Martin R.S., Spence D.M. (2013). A 3D printed fluidic device that enables integrated features. Anal. Chem..

[92] Erkal J.L., Selimovic A., Gross B.C., Lockwood S.Y., Walton E.L., McNamara S., Martin R.S., Spence D.M. (2014). 3D printed microfluidic devices with integrated versatile and reusable electrodes. Lab Chip.

[93] Chudobova D., Cihalova K., Skalickova S., Zitka J., Rodrigo M.A.M., Milosavljevic V., Hynek D., Kopel P., Vesely R., Adam V. (2015). 3D-printed chip for detection of methicillin-resistant Staphylococcus aureus labeled with gold nanoparticles. Electrophoresis.

[94] Krejcova L., Nejdl L., Rodrigo M.A.M., Zurek M., Matousek M., Hynek D., Zitka O., Kopel P., Adam V., Kizek R. (2014). 3D printed chip for electrochemical detection of influenza virus labeled with CdS quantum dots. Biosens. Bioelectron..

[95] Sugioka K., Hanada Y., Midorikawa K. (2008). 3D microstructuring of glass by femtosecond laser direct writing and application to biophotonic microchips. Prog. Electromagn. Res. Lett..

[96] Hanada Y., Sugioka K., Shihira-Ishikawa I., Kawano H., Miyawaki A., Midorikawa K. (2011). 3D microfluidic chips with integrated functional microelements fabricated by a femtosecond laser for studying the gliding mechanism of cyanobacteria. Lab Chip.

[97] Huang T.Q., Qu X., Liu J., Chen S. (2014). 3D printing of biomimetic microstructures for cancer cell migration. Biomed. Microdevices.

[98] Neils C., Tyree Z., Finlayson B., Folch A. (2004). Combinatorial mixing of microfluidic streams. Lab Chip.

[99] Bhargava K.C., Thompson B., Malmstadt N. (2014). Discrete elements for 3D microfluidics. Proc. Natl. Acad. Sci. USA.

[100] Robinson C.D., Auchtung J.M., Collins J., Britton R.A. (2014). Epidemic Clostridium difficile strains demonstrate increased competitive fitness compared to nonepidemic isolates. Infect. Immun..

[101] Lind J.U., Busbee T.A., Valentine A.D., Pasqualini F.S., Yuan H., Yadid M., Park S.J., Kotikian A., Nesmith A.P., Campbell P.H. (2017). Instrumented cardiac microphysiological devices via multimaterial three-dimensional printing. Nat. Mater..

[102] Guan A., Wang Y., Phillips K.S., Li Z. (2016). A contact-lens-on-a-chip companion diagnostic tool for personalized medicine. Lab Chip.

[103] Chapman R.G., Ostuni E., Liang M.N., Meluleni G., Kim E., Yan L., Pier G., Warren H.S., Whitesides G.M. (2001). Polymeric thin films that resist the adsorption of proteins and the adhesion of bacteria. Langmuir.

[104] Zhang H., Hao R., Ren X., Yu L., Yang H., Yu H. (2013). PEG/lecithin–liquid-crystalline composite hydrogels for quasi-zero-order combined release of hydrophilic and lipophilic drugs. RSC Adv..

[105] Goddard J.M., Hotchkiss J.H. (2007). Polymer surface modification for the attachment of bioactive compounds. Prog. Polym. Sci..

[106] Zheng J., Li L., Tsao H.-K., Sheng Y.-J., Chen S., Jiang S. (2005). Strong Repulsive Forces between Protein and Oligo (Ethylene Glycol) Self-Assembled Monolayers: A Molecular Simulation Study. Biophys. J..

[107] Herrwerth S., Eck W., Reinhardt S., Grunze M. (2003). Factors that determine the protein resistance of oligoether self-assembled monolayers-Internal hydrophilicity, terminal hydrophilicity, and lateral packing density. J. Am. Chem. Soc..

[108] Vermette P., Meagher L. (2003). Interactions of phospholipid- and poly(ethylene glycol)-modified surfaces with biological systems: Relation to physico-chemical properties and mechanisms. Colloids Surf. B Biointerfaces.

[109] Morra M. (2000). On the molecular basis of fouling resistance. J. Biomater. Sci. Polym. Ed..

[110] Zhang Z., Wang J., Tu Q., Nie N., Sha J., Liu W., Liu R., Zhang Y., Wang J. (2011). Surface modification of PDMS by surface-initiated atom transfer radical polymerization of water-soluble dendronized PEG methacrylate. Colloids Surf. B Biointerfaces.

[111] Bernards M.T., Cheng G., Zhang Z., Chen S. (2008). Nonfouling Polymer Brushes via Surface-Initiated, Two-Component Atom Transfer Radical Polymerization—Macromolecules (ACS Publications). Macromolecules.

[112] Bernards M., He Y. (2014). Polyampholyte polymers as a versatile zwitterionic biomaterial platform. J. Biomater. Sci. Polym. Ed..

[113] Anthony Yesudass S., Mohanty S., Nayak S.K., Rath C.C. (2017). Zwitterionic–polyurethane coatings for non-specific marine bacterial inhibition: A nontoxic approach for marine application. Eur. Polym. J..

[114] Li G., Xue H., Gao C., Zhang F., Jiang S. (2010). Nonfouling polyampholytes from an ion-pair comonomer with biomimetic adhesive groups. Macromolecules.

[115] Parker A.P., Reynolds P.A., Lewis A.L., Kirkwood L., Hughes L.G. (2005). Investigation into potential mechanisms promoting biocompatibility of polymeric biomaterials containing the phosphorylcholine moiety: A physicochemical and biological study. Colloids Surf. B Biointerfaces.

[116] Murphy E.F., Keddie J.L., Lu J.R., Brewer J., Russell J. (1999). The reduced adsorption of lysozyme at the phosphorylcholine incorporated polymer/aqueous solution interface studied by spectroscopic ellipsometry. Biomaterials.

[117] Yu L., Li C.M., Liu Y., Gao J., Wang W., Gan Y. (2009). Flow-through functionalized PDMS microfluidic channels with dextran derivative for ELISAs. Lab Chip.

[118] Hu S.G., Jou C.H., Yang M.C. (2003). Protein adsorption, fibroblast activity and antibacterial properties of poly(3-hydroxybutyric acid-co-3-hydroxyvaleric acid) grafted with chitosan and chitooligosaccharide after immobilized with hyaluronic acid. Biomaterials.

[119] McArthur S.L., McLean K.M., Kingshott P., St John H.A.W., Chatelier R.C., Griesser H.J. (2000). Effect of polysaccharide structure on protein adsorption. Colloids Surf. B Biointerfaces.

[120] Martwiset S., Koh A.E., Chen W. (2006). Nonfouling characteristics of dextran-containing surfaces. Langmuir.

[121] Zhao C., Li L., Wang Q., Yu Q., Zheng J. (2011). Effect of film thickness on the antifouling performance of poly(hydroxy-functional methacrylates) grafted surfaces. Langmuir.

[122] Wu D., Luo Y., Zhou X., Dai Z., Lin B. (2005). Multilayer poly(vinyl acohol)-adsorbed coating on poly(dimethylsiloxane) microfluidic chips for biopolymer separation. Electrophoresis.

[123] Huang X., Doneski L.J., Wirth M.J. (1998). Surface-Confined Living Radical Polymerization for Coatings in Capillary Electrophoresis. Anal. Chem..

[124] Huang X., Wirth M.J. (1997). Surface-initiated radical polymerization on porous silica. Anal. Chem..

[125] Yu H.J., Luo Z.H. (2010). Novel superhydrophobic silica/poly(siloxane-fluoroacrylate) hybrid nanoparticles prepared via two-step surface-initiated ATRP: Synthesis, characterization, and wettability. J. Polym. Sci. Part A Polym. Chem..

[126] Wang Y., Betts D.E., Finlay J.A., Brewer L., Callow M.E., Callow J.A., Wendt D.E., Desimone J.M. (2011). Photocurable amphiphilic perfluoropolyether/poly(ethylene glycol) networks for fouling-release coatings. Macromolecules.

[127] Sundaram H.S., Cho Y., Dimitriou M.D., Finlay J.A., Cone G., Williams S., Handlin D., Gatto J., Callow M.E., Callow J.A. (2011). Fluorinated amphiphilic polymers and their blends for fouling-release applications: The benefits of a triblock copolymer surface. ACS Appl. Mater. Interfaces.

[128] Martinelli E., Agostini S., Galli G., Chiellini E., Glisenti A., Pettitt M.E., Callow M.E., Callow J.A., Graf K., Bartels F.W. (2008). Nanostructured films of amphiphilic fluorinated block copolymers for fouling release application. Langmuir.

[129] Wyss Institute SLIPS (Slippery Liquid-Infused Porous Surfaces). https://wyss.harvard.edu/technology/slips-slippery-liquid-infused-porous-surfaces.

[130] Wong T.-S., Kang S.H., Tang S.K.Y., Smythe E.J., Hatton B.D., Grinthal A., Aizenberg J. (2011). Bioinspired self-repairing slippery surfaces with pressure-stable omniphobicity. Nature.

[131] Epstein A.K., Wong T.-S., Belisle R.A., Boggs E.M., Aizenberg J. (2012). Liquid-infused structured surfaces with exceptional anti-biofouling performance. Proc. Natl. Acad. Sci. USA.

[132] Vogel N., Belisle R.A., Hatton B., Wong T.S., Aizenberg J. (2013). Transparency and damage tolerance of patternable omniphobic lubricated surfaces based on inverse colloidal monolayers. Nat. Commun..

[133] Juthani N., Howell C., Ledoux H., Sotiri I., Kelso S., Kovalenko Y., Tajik A., Vu T.L., Lin J.J., Sutton A. (2016). Infused polymers for cell sheet release. Sci. Rep..

[134] Kota A.K., Kwon G., Tuteja A. (2014). The design and applications of superomniphobic surfaces. NPG Asia Mater..

[135] Li X.-M., Reinhoudt D., Crego-Calama M. (2007). What do we need for a superhydrophobic surface? A review on the recent progress in the preparation of superhydrophobic surfaces. Chem. Soc. Rev..

[136] Grinthal A., Aizenberg J. (2014). Mobile interfaces: Liquids as a perfect structural material for multifunctional, antifouling surfaces. Chem. Mater..

[137] Kim P., Kreder M.J., Alvarenga J., Aizenberg J. (2013). Hierarchical or not? Effect of the length scale and hierarchy of the surface roughness on omniphobicity of lubricant-infused substrates. Nano Lett..

[138] Maccallum N., Howell C., Kim P., Sun D., Friedlander R., Ranisau J., Ahanotu O., Lin J.J., Vena A., Hatton B. (2015). Liquid-Infused Silicone As a Biofouling-Free Medical Material. ACS Biomater. Sci. Eng..

[139] Howell C., Vu T.L., Johnson C.P., Hou X., Ahanotu O., Alvarenga J., Leslie D.C., Uzun O., Waterhouse A., Kim P. (2015). Stability of surface-immobilized lubricant interfaces under flow. Chem. Mater..

[140] Keller D., Besch W. (2001). Plasma-Induced Surface Functionalization of Polymeric Biomaterials in Ammonia Plasma. Contrib. Plasma Phys..

[141] Badv M., Jaffer I.H., Weitz J.I., Didar T.F. (2017). An omniphobic lubricant-infused coating produced by chemical vapor deposition of hydrophobic organosilanes attenuates clotting on catheter surfaces. Sci. Rep..

[142] Milton Harris J., Chess R.B. (2003). Effect of pegylation on pharmaceuticals. Nat. Rev. Drug Discov..

[143] Sotiri I., Overton J.C., Waterhouse A., Howell C. (2016). Immobilized liquid layers: A new approach to anti-adhesion surfaces for medical applications. Exp. Biol. Med..

[144] Zhang F., Sautter K., Larsen A.M., Findley D.A., Davis R.C., Samha H., Linford M.R. (2010). Chemical vapor deposition of three aminosilanes on silicon dioxide: Surface characterization, stability, effects of silane concentration, and cyanine dye adsorption. Langmuir.

[145] Liu Z.Z., Wang Q., Liu X., Bao J.Q. (2008). Effects of amino-terminated self-assembled monolayers on nucleation and growth of chemical vapor-deposited copper films. Thin Solid Films.

[146] Baker J., Stephenson T., Dard S., Cote P. (1995). Characterisation of fouling of nanofiltration membranes used to treat surface waters. Environ. Technol..

[147] Van Paassen J.A.M., Kruithof J.C., Bakker S.M., Kegel F.S. (1998). Integrated multi-objective membrane systems for surface water treatment: Pre-treatment of nanofiltration by riverbank filtration and conventional ground water treatment. Desalination.

[148] Cornelissen E.R., Vrouwenvelder J.S., Heijman S.G.J., Viallefont X.D., Van Der Kooij D., Wessels L.P. (2007). Periodic air/water cleaning for control of biofouling in spiral wound membrane elements. J. Membr. Sci..

[149] Majamaa K., Aerts P.E.M., Groot C., Paping L.L.M.J., van den Broek W., van Agtmaal S. (2010). Industrial water reuse with integrated membrane system increases the sustainability of the chemical manufacturing. Desalin. Water Treat..

[150] Miller D.J., Araújo P.A., Correia P.B., Ramsey M.M., Kruithof J.C., van Loosdrecht M.C.M., Freeman B.D., Paul D.R., Whiteley M., Vrouwenvelder J.S. (2012). Short-term adhesion and long-term biofouling testing of polydopamine and poly(ethylene glycol) surface modifications of membranes and feed spacers for biofouling control. Water Res..

[151] Ronen A., Resnick A., Lerman S., Eisen M.S., Dosoretz C.G. (2015). Biofouling suppression of modified feed spacers: Localized and long-distance antibacterial activity. Desalination.

[152] Ronen A., Lerman S., Ramon G.Z., Dosoretz C.G. (2015). Experimental characterization and numerical simulation of the anti-biofuling activity of nanosilver-modified feed spacers in membrane filtration. J. Membr. Sci..

[153] Wibisono Y., Yandi W., Golabi M., Nugraha R., Cornelissen E.R., Kemperman A.J.B., Ederth T., Nijmeijer K. (2015). Hydrogel-coated feed spacers in two-phase flow cleaning in spiral wound membrane elements: Anovel platform for eco-friendly biofouling mitigation. Water Res..

[154] Araújo P.A., Kruithof J.C., Van Loosdrecht M.C.M., Vrouwenvelder J.S. (2012). The potential of standard and modified feed spacers for biofouling control. J. Membr. Sci..

[155] McCullough E.J., Yadavalli V.K. (2013). Surface modification of fused deposition modeling ABS to enable rapid prototyping of biomedical microdevices. J. Mater. Process. Technol..

[156] Upcraft S., Fletcher R. (2003). The rapid prototyping technologies. Assem. Autom..

